# Acceptability, feasibility, and user satisfaction of a virtual reality relaxation intervention in a psychiatric outpatient setting during the COVID-19 pandemic

**DOI:** 10.3389/fpsyt.2023.1271702

**Published:** 2023-10-25

**Authors:** Annika Humbert, Elisabeth Kohls, Sabrina Baldofski, Carola Epple, Christine Rummel-Kluge

**Affiliations:** ^1^Department of Psychiatry and Psychotherapy, Medical Faculty, Leipzig University, Leipzig, Germany; ^2^Department of Psychiatry and Psychotherapy, Leipzig University Medical Center, Leipzig, Germany; ^3^Lab E GmbH, Esslingen, Germany

**Keywords:** virtual reality, relaxation, feasibility, psychiatric outpatients, mental disorder

## Abstract

**Background:**

The COVID-19 pandemic was particularly difficult for individuals with mental disorders. Due to governmental restrictions, face-to-face offers for psychiatric outpatients like therapies, psychoeducational groups or relaxation courses were limited. Virtual reality (VR) might be a new possibility to support these patients by providing them with a home-based relaxation tool.

**Objective:**

The aim of this study was to evaluate the acceptability, feasibility, and user satisfaction of a supportive therapy-accompanying, relaxation VR intervention in psychiatric outpatients during the COVID-19 pandemic in Germany.

**Methods:**

The four-weeks VR intervention consisted of regular watching of relaxing videos in the participants’ home environment. Sociodemographics, feasibility (frequency of use, user-friendliness), satisfaction (Client Satisfaction Questionnaire-8), depressive symptoms (Patient Health Questionnaire-9), quality of life (abbreviated World Health Organization Quality of Life assessment), and credibility and expectancy (Credibility Expectancy Questionnaire-8) were measured in an intention-to-treat (ITT) analysis and a per-protocol (PP) analysis of completers.

**Results:**

In total, *N* = 40 patients participated in the study. Most of the participants in the ITT analysis (*n* = 30, 75.0%) used the VR device three or 4 weeks. A majority of the *N* = 29 completers (PP: *n* = 18, 62.1%) used it all 4 weeks. Most participants used the device two or more times a week (ITT: *n* = 30, 83.3%; PP: *n* = 26, 89.7%) and described the user-friendliness as rather or very easy (ITT: *n* = 33, 91.7%; PP: *n* = 26, 89.7%). User satisfaction was high (ITT: 19.42, SD = 4.08; PP: M = 20.00, SD = 4.19) and did not correlate with participants’ sex or age (all *p* < 0.05). Depressive symptoms and psychological quality of life improved significantly from pre-to post-intervention (ITT and PP, all *p* < 0.05). Higher pre-intervention credibility significantly correlated with a better outcome of satisfaction (ITT and PP), depressive symptoms, physical, psychological, and social quality of life (PP; all *p* < 0.05).

**Conclusion:**

A supportive therapy-accompanying VR relaxation intervention is feasible and acceptable in a psychiatric outpatient setting. Due to the high satisfaction and user-friendliness, VR can be an easy to implement relaxation tool to support psychiatric outpatients.

**Clinical trial registration:**

https://clinicaltrials.gov/, DRKS00027911.

## 1. Introduction

The outbreak of the COVID-19 pandemic had a serious impact on peoples’ mental health around the globe (1). As shown by previous research, individuals with already existing mental disorders are particularly vulnerable facing the pandemic situation (2–4). Problems like limited access to mental health services, social isolation, and disruptions to daily routine were shown to have a negative impact on mental health outcomes of these individuals (2). Furthermore, health anxiety and fear of the virus are likely to increase the risk of symptom deterioration and crises among individuals with mental disorders (5). Due to governmental restrictions to stop the spread of the virus, many mental health care providers had to reduce their face-to-face offers for patients. Therefore, psychiatric outpatients who otherwise received treatment in therapies, psychoeducational groups or relaxation courses several times a week, not only lost part of their therapeutic support, but also their necessary daily structure.

To face these challenges and continue providing mental health care at the height of COVID-19, an urgent need of new treatment options preferable from the field of e-mental health arose (6). One of these options might be the use of virtual reality (VR), a computer-generated simulation of a real environment which can be explored and interacted with by a person using a special VR headset (7). During the last decade VR has become a major area of interest within the modern health community (8). It has been shown to have potential as a supportive and therapeutic tool for patients with different mental disorders (7–10). Numerous studies established the efficacy of VR for behavioral exposure therapy in patients with anxiety disorders, such as phobia or post-traumatic stress disorder (PTSD) (11–15). Some positive results were also shown in the treatment of obsessive-compulsive disorder (OCD) (16, 17) and substance use disorders (18–20). While there are multiple studies using VR interventions for patients with mental disorders, comparably little has been published on the use of VR for patients with depression (21, 22). Even though our study followed a transdiagnostic approach, patients with depression made up a large part of the sample.

Previous studies indicated that exposure to a natural environment using VR reduced stress levels and improved mood in healthy individuals (23–25). Further, it is known that various techniques promoting relaxation are beneficial in the treatment of psychiatric patients and can reduce depressive symptoms and anxiety (26, 27).

A small number of randomized controlled studies examined the effect of watching 360-degree VR nature videos in adults with mental disorders (28–30). Most of these studies used immersive 360-degree VR technology, i.e., 360-degree VR videos were viewed *via* head-mounted-displays that recognized the users’ head movement, so users were able to see the natural environment all around them. The immersive effect created by this technology provides the feeling of really being present in the natural environment displayed in the videos. The use of 360-degree VR videos and the resulting immersion into real natural environments showed greater effects on stress and negative affect compared to urban VR environments or two-dimensional nature videos (31, 32).

Some studies even went a step further by using interactive VR. Since users can interact with the environment in this approach, it is possible that they feel more present and reach a greater extent of relaxation (33). However, it has also been shown that interactive environments may be distractive and lead to less attention and engagement (34). The results of these previous studies indicated that relaxing VR is in general feasible and acceptable and had a positive effect on mood, stress levels, and psychiatric symptoms.

There are already some studies and case studies who investigated the use of home-based and self-led VR interventions to support individuals with various mental health conditions (28, 35–37). To the best of our knowledge there are to date no studies that investigated more specifically if the implementation of a home-based VR relaxation intervention using 360-degree nature videos is feasible, satisfactory and acceptable for psychiatric outpatients with different mental disorders. In this study psychiatric outpatients were patients with particularly severe mental disorders who could not be adequately treated by resident psychiatrist or psychotherapists. Due to the pandemic situation, the possibility to offer those patients a self-manageable relaxation tool which they can use independently from governmental restrictions in their home environment appeared promising, advantageous, and adequate.

The aim of this study was to assess the acceptability, feasibility, and user satisfaction of a supportive therapy-accompanying VR intervention in a psychiatric outpatient setting, where individuals with particularly severe mental disorders receive treatment. The VR intervention was developed during the lockdown phase of the COVID-19 pandemic and had been implemented in the participants’ home environment over 4 weeks. We hypothesized that the implementation of VR would be acceptable, feasible and satisfactory in that patient group. A specification of this hypothesis can be found under materials and methods.

In addition, it was evaluated if the satisfaction was related to the participants sex, age or depressive symptoms, and if there was a change in depressive symptoms and quality of live before and after the intervention.

## 2. Materials and methods

### 2.1. Study design

**Figure 1:**
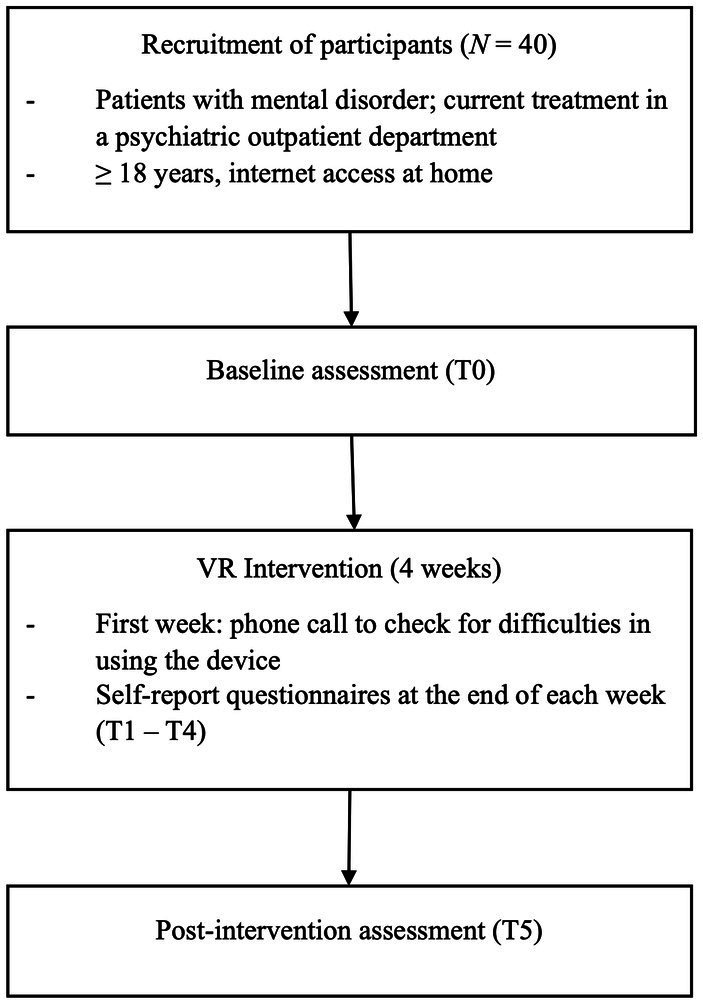
Flowchart of trial design.

This observational study evaluated a VR relaxation intervention in a psychiatric outpatient setting. A flowchart of the trial design is displayed in Figure 1. The study was approved by the ethics committee of the Medical Faculty of the University of Leipzig (275/21-ek, 09/2021). It was registered in the German Clinical Trial Register (DRKS00027911).

### 2.2. Participants and recruitment

The recruitment took place from January 2022 until July 2022. Participants were psychiatric outpatients currently receiving treatment in the psychiatric outpatient department of the Leipzig University Medical Center, in Germany. In this department, people with severe mental disorders who are not reached by resident psychiatrists or psychotherapists receive medical and psychotherapeutic treatment.

When the recruitment started in January 2022, various governmental restrictions to reduce the spread of COVID-19 in Germany were in place (e. g., prohibition of meetings with more than 10 people, requirement of vaccination or full recovery status and negative COVID-19 test result for entering public places). These restrictions were mostly ongoing until the end of recruitment in July 2022 and affected the therapeutic offers of the psychiatric outpatient department of the Leipzig University Medical Center to a huge extent, including the access for patients to therapies, psychoeducational groups, and relaxation courses.

Inclusion criteria were current treatment in the psychiatric outpatient department, age of 18 years or older, adequate knowledge of the German language, sufficient sight and reading ability, and internet access at home. Exclusion criteria were the diagnoses schizophrenia, schizoaffective disorder, and bipolar affective disorder (based on medical records). Patients with the diagnoses schizophrenia and schizoaffective disorder were excluded because one of the main symptoms of these severe mental disorders are hallucinations. In this case it could be dangerous to confront patients with additional visual and auditory stimuli that especially in VR seem particularly real. Patients with the diagnosis bipolar affective disorder were excluded because for patients with acute mania a reduction of stimuli is necessary and we did not want to risk that an unguided use at home would counteract this. Due to the homebased implementation of the intervention, it was considered too risky to include patients with these three diagnoses.

Further we decided to exclude patients with pre-existing severe motion sickness (based on self-report). Due to results of previous studies showing that a motion sickness history in real life is an important predictor for the experience of cyber sickness (38, 39), self-reported motion sickness is a common exclusion criterion in VR studies (40).

Patients were informed about the VR intervention by their psychiatrists or psychotherapists, or *via* the study information placed in the waiting area at the department. Referring psychotherapists and psychiatrists were all medical personnel working in the same outpatient department where the study was conducted. Therefore, no compensation was provided for them.

Patients who were interested in the study were screened regarding the inclusion and exclusion criteria via telephone. If they were suitable for participation in the study, they were invited to a face-to-face appointment in the outpatient department. Prior to this appointment they were sent an information sheet about the study procedure via e-mail.

During the face-to-face appointment, participants were further informed about the study and provided written informed consent prior to study participation. They filled in the baseline (T0) paper-and-pencil questionnaires. Additionally, participants received a detailed introduction into the home use of the VR device. Participants were first explained in words and by going through a manual sheet how to turn on the VR device and start a video step by step (i.e., press on-button, establish wi-fi connection, open VR application, choose category and video). Afterwards, participants completed all the steps at least once together with one of the researchers. VR device and manual sheet were sent home with the participant after the introduction meeting.

### 2.3. Virtual reality intervention

**Figure 2:**
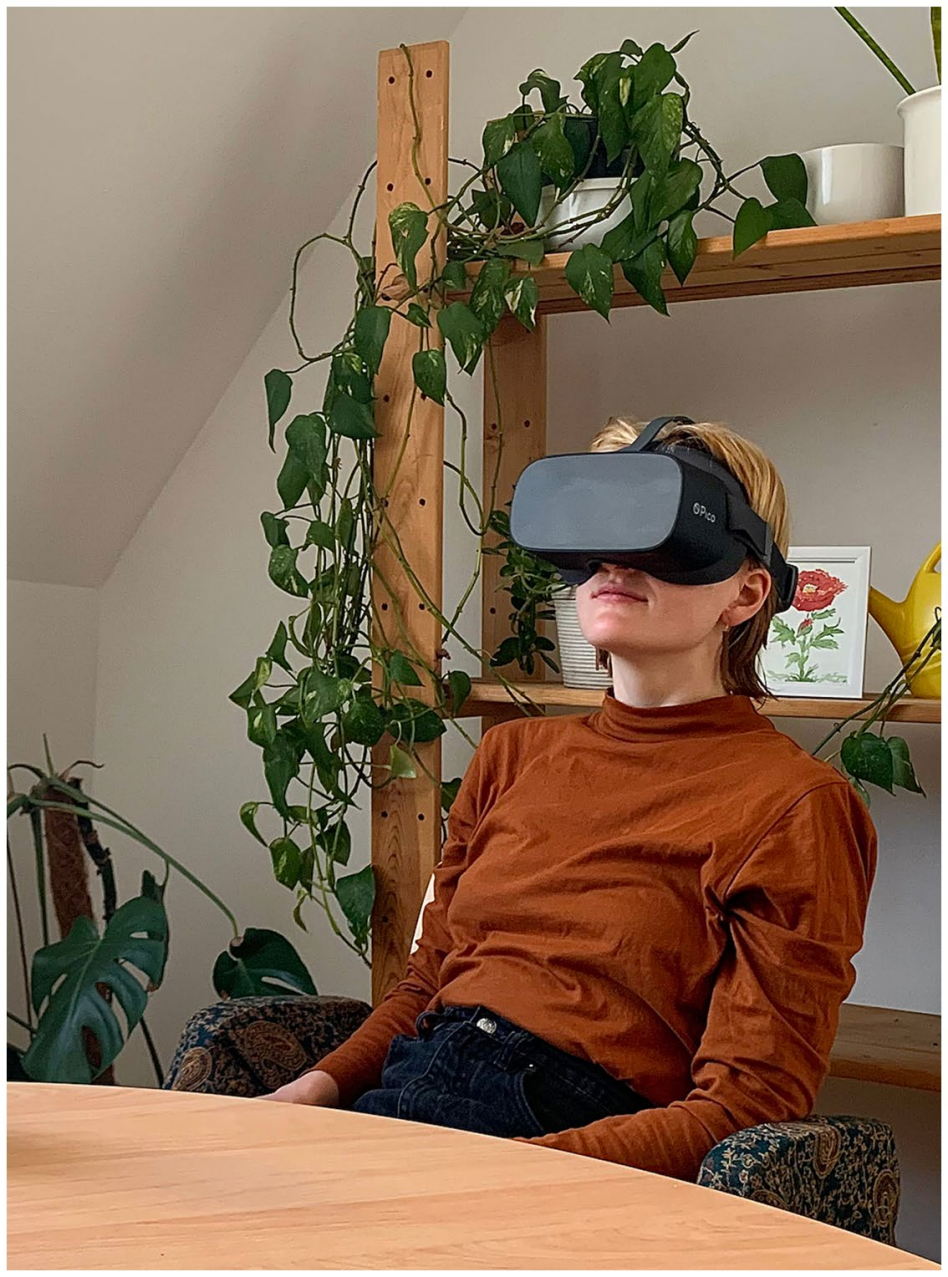
VR device in use (please note the picture is showing the first author, AH, and is published with consent).

The intervention consisted of regular watching of different relaxing 360-degree VR nature videos. Devices used in this study were the stand-alone VR headset ‘Pico G2 4K’, a high-quality product with a 4 K LCD display with 3,840 × 2,160 screen resolution, which were rented by the research team for the conduct of the study (please see Figure 2 for a picture of the VR device in use). Use of the device was free-of-charge for participants.

Participants were instructed to watch the videos with the VR device in their home environment over a period of 4 weeks. It was recommended to use the VR device at least two times a week for about 20 min at a time. Participants were free to choose the days and times when they would watch the videos. They could also freely choose the videos they wanted to watch out of a video library of 20 videos at each occasion. During the first week of the intervention, all participants received a phone call to check if there were any difficulties using the VR device and to give further advice if necessary.

After turning on the VR device videos could be started by opening an application which was preinstalled on the device. The VR videos were produced by Lab E GmbH and the application was designed by TwinC GmbH. Both the application and videos already existed before the study and videos were provided free of charge by Lab E GmbH for the purpose of this study. Provision of videos was not accompanied by any obligations or agreements between Lab E GmbH and the research team. Lab E GmbH is specialized on producing VR videos for therapeutic purposes and offers courses on the use of VR in therapeutic settings. The videos have been in clinical use by practicing psychotherapists for several years.

**Figure 3:**
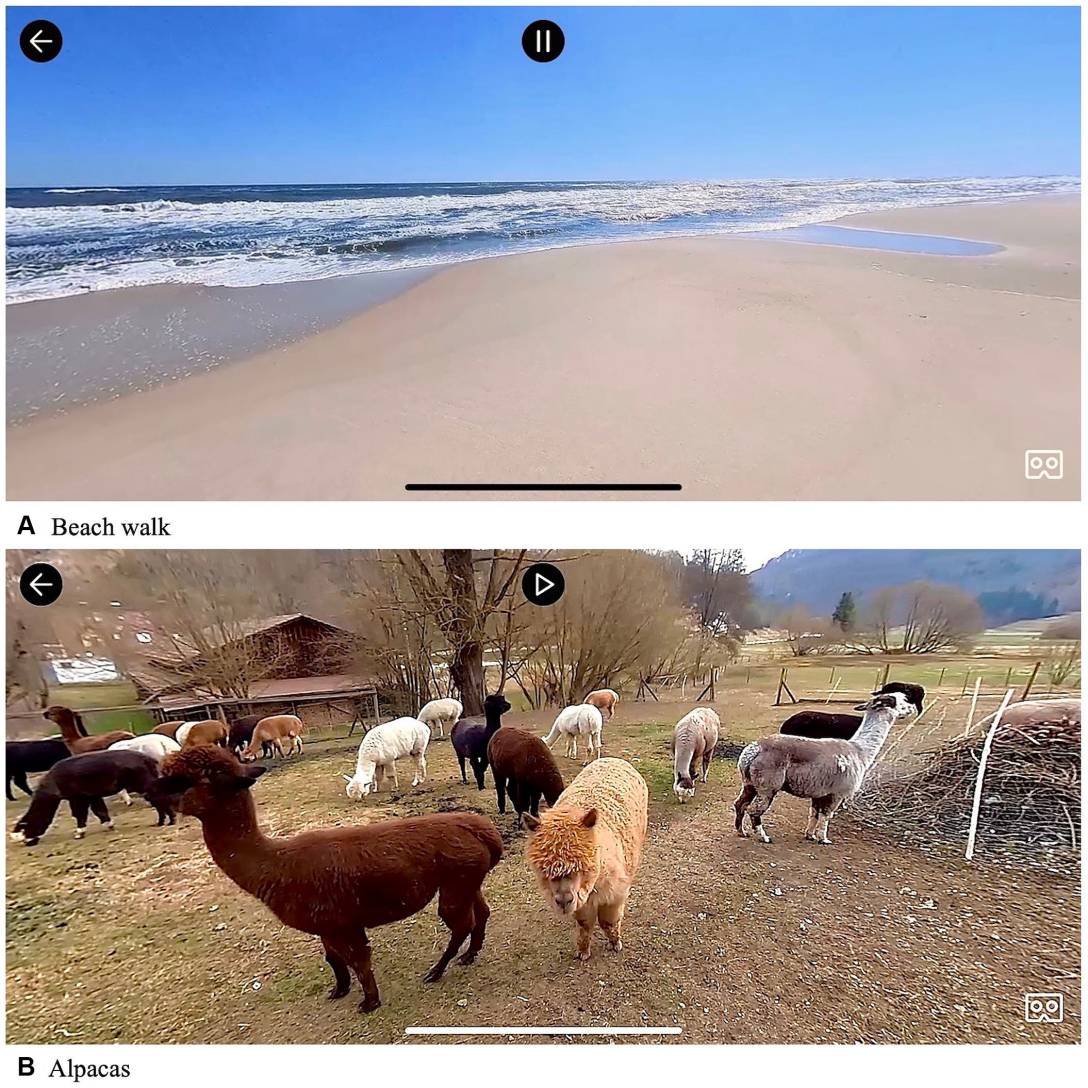
Screenshots of VR videos.

Prior to conduction of the study, 20 videos were selected that met certain relaxation criteria: First, their visual content should consist of a peaceful and quiet scenery of real natural environment that should be low in stimuli, and second, they should either include real nature sounds or relaxation music. Further, the user interface of the application was adapted for the purpose of this intervention, so that participants could only choose between the selected 20 videos. The adapted user interface consisted of four different categories, each of them containing between four and six videos that displayed different natural environments. Categories included ‘walks’ (e. g., ‘river walk’, ‘forest walk’, ‘beach walk’), ‘landscapes’ (e. g., ‘forest’, ‘wheat field’, ‘beach’), ‘animals’ (e. g., ‘horses’, ‘rabbits’, ‘alpacas’), and ‘snow’ (e. g., ‘snowy forest’, ‘waterfall’; please see Figure 3 for screenshots of example videos).

All videos included different relaxing elements which can be divided into visual and auditory stimuli. Regarding visual stimuli, all videos had in common that participants were able to observe the real-time scenery of nature with a 360-degree view and thus, had the possibility to immerse into the virtual environment. The visual content of the videos was comparable, with the only exception being that in the videos of the category ‘walks’, the watchers’ position in the video was moving and the environment was changing, while in all other videos the watchers’ position in the video was still. For the safety of participants, all videos were explicitly designed to be watched while being seated, so that participants did not have to stand or walk around while using the VR glasses.

Regarding auditory stimuli, videos had two different types of relaxing sound stimuli. Most of the videos (*n* = 12, 60.0%) were accompanied by their original nature sounds, while the other videos (*n* = 8, 40.0%) were accompanied by relaxation music.

The videos each lasted between 6 and 29 min, so participants had the option of watching, i.e., only one longer video or multiple short videos in each 20-min session.

All participants were informed that the VR intervention was a supportive offer of the outpatient department to evaluate the acceptability, feasibility, and user satisfaction, and no replacement of their regular therapies.

### 2.4. Completers and dropouts

Participants who used the VR device at least three out of 4 weeks (based on self-report) and completed all questionnaires were classified as completers. Participants who used the VR device less than 3 weeks (based on self-report) or did not complete all questionnaires were classified as dropouts. Present data of all study participants (completers and dropouts) was evaluated in an intention-to-treat (ITT) analysis, while data of completers was evaluated in a per-protocol (PP) analysis. Further, all dropouts who used the VR device less than 3 weeks were followed-up via telephone after the intervention to assess reasons for non-completion.

### 2.5. Measures

Self-report questionnaires (paper-and-pencil in a booklet) were filled in by participants at baseline (pre-intervention, T0), at the end of week 1, 2, 3 and 4 of the intervention (T1–T4), and post-intervention (T5; see Table 1). The maximum time between the end of the 4-week intervention (T4) and T5 was 1 week, due to organizational reasons.

**Table 1:** Study measures (self-report questionnaires).

Instrument	Timepoint		
	T0	T1 – T4	T5
Sociodemographics (7 items) and Diagnoses^1^	X		
Use of VR Device and Reason for Non-use (1 item)		X	
Videos watched (1 item)		X	
Frequency of use (1 item)			X
User-friendliness (2 items)			X
Best liked videos (1 item)			X
Client Satisfaction Questionnaire-8 (ZUF-8; 2 items)		X	
Client Satisfaction Questionnaire-8 (ZUF-8; 7 items)			X
Patient Health Questionnaire-9 (PHQ-9)	X		X
WHO Quality of Life-BREF (WHOQOL-BREF)	X		X
Credibility Expectancy Questionnaire (CEQ)	X		X

^1^Assessed based on medical records.

#### 2.5.1. Sociodemographics and diagnoses

At baseline (T0), participants filled in a questionnaire about basic sociodemographic characteristics (age, sex, marital status, living situation, parenthood, and employment status). Further, they were asked about their attitudes toward the pandemic situation. In addition to the self-report questionnaire, participants’ diagnoses were assessed based on medical records of their therapists.

#### 2.5.2. Videos

After each week (T1–T4), participants had to mark in a list of all videos which videos they had watched in the previous week. After the intervention (T5), participants were asked to name three videos they liked best.

#### 2.5.3. Feasibility

After each week (T1–T4), participants had to indicate in a questionnaire if they had used the VR device in the previous week. If they did not use the device in that week, they were further asked to specify a reason for non-use (no time, no interest, technical problems, or other reasons). After the intervention (T5), participants were asked how they would describe the user-friendliness of the VR device and the application on a 4-point Likert scale (1 = very difficult, 2 = rather difficult, 3 = rather easy, 4 = very easy). For the purpose of this analysis response option 3 and 4 of this item were summarized as ‘rather or very easy’. Further, participants were asked how long they needed from starting the device to start watching a video on a 4-point Likert scale from 1 = more than 10 minutes to 4 = less than 3 minutes.

#### 2.5.4. Satisfaction

The evaluation of participants’ satisfaction was based on the ZUF-8, which is the German version of the Client Satisfaction Questionnaire-8 (CSQ-8) (41). At the end of each week of the intervention (T1–T4), participants answered two adapted items of the ZUF-8 to assess their satisfaction with the VR videos they watched during the previous week. To answer those items, they had to indicate on a 4-point Likert scale from 1 = definitely no to 4 = definitely yes, if the videos helped them to relax and/or had a positive benefit on their psychological well-being, and if they would recommend the intervention with these videos to a friend.

Post-intervention (T5), participants were asked to fill in a 7-item satisfaction questionnaire, based on the ZUF-8. For this adapted 7-item version of the ZUF-8 six of the original items were retained and slightly rephrased, and one item about immersion into the virtual environment was added. The wording of the six retained items was adapted to the VR intervention as in the following example: A question like ‘How would you rate the quality of service you received?’ was changed to ‘How would you rate the quality of the VR videos that you watched?’. Each item was rated on a 4-point Likert scale from 1 = lowest satisfaction to 4 = highest satisfaction. For the individual evaluation of the items of ZUF-8, response options 3 and 4 were summarized as ‘definitely or generally yes’ or ‘somehow or a lot’.

The overall satisfaction with the intervention was measured by a total sum score ranging from 7 to 28, with higher scores representing higher satisfaction. As in several studies using the CSQ-8 a score of ≥20 (out of 32) points was interpreted as acceptable or moderate satisfaction (42, 43), we transferred this to our 7-item version and thus, a mean sum score of 17.5 was defined as a cut off for satisfaction. Previous research indicated that the ZUF-8 had a high reliability and internal consistency (41, 44).

#### 2.5.5. Depressive symptoms

At baseline (T0) and post-intervention (T5), depressive symptoms were assessed with the Patient Health Questionnaire-9 (PHQ-9) (45–47). The PHQ-9 consists of nine items measuring different depressive symptoms on a 4-point Likert scale from 0 = not at all to 3 = nearly every day. A total sum score, ranging from 0 to 27, was calculated, with higher scores representing higher levels of depressive symptoms. In addition, sum scores were classified to represent different levels of severity of depressive symptoms from minimal, 0 to 4, mild, 5 to 9, moderate, 10 to 14, moderately severe, 15 to 19, to severe, 20 to 27, 47). Good reliability, validity, and internal consistency of the PHQ-9 have been shown in former studies (45).

#### 2.5.6. Quality of life

The participants’ quality of life was measured with a short version of the World Health Organization Quality of Life assessment WHOQOL-BREF (48, 49); at baseline (T0) and post-intervention (T5). The WHOQOL-BREF consists of 26 items to assess four different domains of quality of life: physical, psychological, social, and environmental quality of life. Items were measured on a 5-point Likert scale from 1 = not at all to 5 = extremely. For each of the four domains of quality of life an index score, ranging from 0 to 100, was calculated. Higher index scores are indicating a higher quality of life. The WHOQOL-BREF has shown a high reliability (49).

#### 2.5.7. Credibility and expectancy

Credibility and expectancy related to the intervention were assessed by the Credibility Expectancy Questionnaire (CEQ) (50) at baseline (T0) and post-intervention (T5). The questionnaire was translated by using a back-translation procedure. The wording of the items was adapted to the VR intervention as in the following example: A question like ‘How logical does the therapy offered to you seem at this point of time?’ was changed to ‘How logical does the virtual reality relaxation tool offered to you seem at this point of time?’

The CEQ consists of six items, three items to measure a credibility factor and three items to measure an expectancy factor. The three items of the credibility factor as well as item number 2 of the expectancy factor were rated on a scale from 1 = lowest expectancy to 9 = highest expectancy, while items number 1 and 3 of the expectancy factor were rated on an 11-point scale from 0% = lowest credibility to 100% = highest credibility. The latter scales were transformed into scales from 1 to 9 to compute sum scores, resulting in two sum scores for credibility and expectancy, respectively, with a range from 3 to 27 for each score. Higher sum scores are representing higher credibility or expectancy. As there are no pre-defined norm scores for the CEQ, for the purpose of this analysis the 70th percentile, corresponding to a sum score of ≥19.5, was defined as a cut-off value for a high credibility and expectancy, respectively. The CEQ has been shown to have a high test–retest reliability and an adequate internal consistency (50).

#### 2.5.8. Follow-up survey of dropouts

The follow-up survey was performed *via* telephone after the intervention was completed for all participants who used the VR device less then 3 weeks. A questionnaire consisting of two items was filled in by the interviewer during the phone call. Specifically, participants were asked in the first item after how many weeks of use (0, 1 or 2 weeks) they stopped using the device. In the second item, they had to indicate their main reasons for non-use (no time, no interest, no motivation, technical problems, or side effects).

### 2.6. Statistical analysis

First, descriptive statistics were computed for all study variables (sociodemographics: age, sex, marital status, living situation, parenthood, employment status, attitudes toward the pandemic situation, diagnoses; feasibility: use/non-use, frequency of use, user-friendliness; videos: watched videos, best liked videos; satisfaction; depressive symptoms; quality of life; credibility/expectancy; reasons for non-use) in the total study sample (ITT analysis) as well as in the subgroup of completers (PP analysis). Additionally, descriptive statistics were computed for the variables weeks of use and reasons of non-use in the group of dropouts.

For reliability analysis, Cronbach’s alpha was calculated to assess the internal consistency of the CEQ (T0 and T5) and the ZUF-8 (T5).

Second, potential differences between completers and dropouts regarding age were tested with a *t*-test for unpaired samples. Chi-square tests were used to compare completers and dropouts on categorical variables (sex, marital status, living situation, employment status, and diagnoses). For these tests, it is of note that some cell frequencies for the variables sex, marital status, and diagnosis were below five, which might impair interpretability of the results based on these variables.

Third, in the ITT and the PP analysis potential differences in satisfaction (ZUF-8) between women and men were tested with a *t*-test for unpaired samples.

Fourth, in the ITT and PP analyzes, *t*-tests for paired samples were performed to analyze differences between baseline (T0) and post-intervention scores (T5) of depressive symptoms (PHQ-9), quality of life (WHOQOL-BREF) and credibility/expectancy (CEQ). In the PP analysis, when analyzing differences in the domains of psychological and social quality of life (WHOQOL-BREF) between baseline (T0) and post-intervention (T5), Wilcoxon tests for paired groups were performed due to non-normal distribution of these variables (as indicated by Shapiro-Wilks test, *p <* 0.05).

Finally, in the ITT and PP analysis Pearson correlation coefficients were used to exploratively analyze correlations between variables. Specifically, correlations between participants’ age and satisfaction, between depressive symptoms at baseline (T0)/post-intervention (T5) and satisfaction, between credibility/expectancy at baseline (T0) and satisfaction, and between credibility/expectancy at baseline (T0) and quality of life post-intervention (T5) were computed.

In the PP analysis, correlations between credibility/expectancy at baseline (T0) and the domains of psychological and social quality of life post-intervention (T5), Spearman correlation coefficients were used due to non-normal distribution (as indicated by Shapiro-Wilks test, *p <* 0.05).

All effect sizes were interpreted as recommended by Cohen. Effect sizes for unpaired *t*-tests (Cohen’s *d*) and paired t-tests (Cohen’s *d_z_*) were interpreted as small, | *d, d_z_* | < 0.50, medium, | *d, d_z_* | ≥ 0.50, and large, | *d, d_z_* | ≥ 0.80 (51). Effect sizes for Wilcoxon tests were interpreted as small, | *r* | < 0.30, medium, | *r* | ≥ 0.30, and large, | *r* | ≥ 0.50 (51). To estimate effect sizes for chi-square tests, the φ coefficient was used, while Cramér’s V (φc) was used when the contingency table was larger than 2×2, with | φ, φc | < 0.30 indicating a small, | φ, φc | ≥ 0.30 a medium, and | φ, φc | ≥ 0.50 a large effect (51). Correlations were interpreted using Pearson (*r*) and Spearman (ρ) correlation coefficients as weak, | *r*, ρ | ≥ 0.10, moderate, | *r*, ρ | ≥ 0.30, or strong, | *r,* ρ | ≥. 50 (51).

Based on the reported results of one main outcome measure (i.e., psychological quality of life), a *post hoc* power analysis was computed, indicating that *n* = 36 participants were sufficient to obtain 83.1% power to detect differences in scores of psychological quality of life when employing a two-tailed *α* = 0.05.

All statistical analyzes were performed using SPSS 27.0 (IBM Corporation). All testing was two-tailed at an α level of 0.05.

### 2.7. Operationalization of acceptability, feasibility, and satisfaction

The study hypothesis was based on three parts: acceptability, feasibility, and user satisfaction. A high feasibility was considered to be present if (1) the intervention would be carried out as recommended by the majority of participants, i.e., the VR device would be used at least 3 out of 4 weeks, and (2) all participants would be able to use the glasses on their own at home and the user friendliness would be considered by the majority of participants as rather or very easy.

Further, the intervention would be considered highly satisfactory if all of the following three aspects are fulfilled: (1) a mean sum of ≥17.5 would be achieved in the adapted ZUF-8, (2) a majority of participants would indicate after each week (T1–T4) and post-intervention (T5) that they would generally or definitely recommend the VR intervention to a friend, and (3) a majority of participants would indicate after each week (T1–T4) and post-intervention (T5) that VR videos helped them somehow or a lot to relax and/or had a positive benefit on their psychological well-being.

Acceptability is a necessary condition of an intervention to be successfully implemented (52). It is already known from former studies that patients are more likely to adhere to treatment recommendations and to benefit from improved clinical outcomes if an intervention is considered acceptable (53, 54). Further studies have inferred directly from the satisfaction with an intervention to its acceptability (42, 43). Therefore, if the VR intervention in this study would be satisfactory and feasible according to the specifications above, it would also be considered acceptable.

## 3. Results

### 3.1. Sample characteristics

In total, *N* = 40 participants were included in the study (ITT analysis). Of these, *n* = 29 (72.5%) participants were classified as completers (PP analysis) and *n* = 11 (27.5%) as dropouts. Of those dropouts, *n* = 7 (63.6%) finished all questionnaires, but used the VR device for less than 3 weeks, and *n* = 4 (36.4%) did not complete all questionnaires. So, in the ITT analysis the data of all *N* = 40 participants could be analyzed regarding T0, while due to missing data, only the data of *n* = 36 (90.0%) could be analyzed regarding T1–T5.

Participants of the study showed an age range of 20–67 years with a mean age of 40.90 (SD = 13.46) in the ITT and 41.62 (SD = 14.00) in the PP analysis. Around two-thirds of the participants in the ITT as well as in the PP analysis were female and one third was male (see Table 2). Most participants currently received treatment for the diagnoses unipolar depression, anxiety disorder or OCD (ITT: *n* = 30, 75.0%; PP: *n* = 23, 79.3%; see Table 2). In both analyzes majority of participants was married or in a relationship and lived together with other people (see Table 2). Approximately half of the participants in both analyzes were currently employed or went to school/university (ITT: *n* = 21, 52.5%; PP: *n* = 15, 51.7%), while the other half were currently either unemployed, incapacitated for work, or retired due to age or illness (ITT: *n* = 19, 47.5%; PP: *n* = 14, 48.3%; see Table 2).

**Table 2:** Sample characteristics of the ITT^1^ and PP^2^ analysis.

Variable	ITT (*n* = 40)	PP (*n* = 29)
Sex, *n* (%)		
Female	28 (70.0)	20 (69.0)
Male	12 (30.0)	9 (31.0)
Age, *M* (*SD*)	40.90 (13.46)	41.62 (14.00)
Marital Status, *n* (%)		
Married/in a relationship	22 (55.0)	20 (69.0)
Single	18 (45.0)	9 (31.0)
Diagnosis, *n* (%)		
Unipolar depression	13 (32.5)	11 (37.9)
Anxiety disorder	9 (22.5)	5 (17.2)
OCD^3^	8 (20.0)	7 (24.1)
ADHD^4^	5 (12.5)	4 (13.8)
Personality disorder	3 (7.5)	2 (6.9)
Other	2 (5.0)	0 (0.0)
Living situation, *n* (%)		
Together with others	29 (72.5)	24 (82.8)
Alone	11 (27.5)	5 (31.0)
Parenthood		
Children	17 (42.5)	13 (44.8)
No children	23 (57.5)	16 (55.2)
Employment Status, *n* (%)		
Employed	13 (32.5)	9 (31.0)
School	1 (2.5)	1 (3.4)
University	7 (17.5)	5 (17.2)
Unemployed	5 (12.5)	3 (10.3)
Currently incapacitated for work	5 (12.5)	4 (13.8)
Retired due to age or illness	9 (22.5)	7 (24.1)

Calculation of % from valid cases of the ITT and PP analysis. ^1^ITT, Intention-to-treat. ^2^PP, Per-protocol. ^3^OCD, Obsessive compulsive disorder. ^4^ADHD, Adult attention-deficit/hyperactivity disorder.

Regarding attitudes toward the pandemic, half of the participants (ITT: *n* = 21, 52.5%; PP: *n* = 15, 51.7%) indicated that they were strongly or very strongly mentally stressed due to the pandemic situation, and *n* = 22 (55.0%) in the ITT and *n* = 10 (34.5%) of the PP analysis indicated that they felt restricted due to governmental regulation.

#### 3.1.1. Comparison of completers and dropouts

Statistically significant differences between completers (PP analysis) and dropouts emerged in the variables marital status, *χ*^2^(1) = 8.31, *p* = 0.004, φ = −0.46 (medium effect), and living situation, *χ*^2^(1) = 5.57, *p* = 0.018, φ = −0.37 (medium effect). Compared to completers, dropouts were more likely to be single (*n* = 9, 81.8%) and to live alone (*n* = 6, 54.4%). There were no significant differences between completers and dropouts in the variables age, *t*(20.46) = −0.58, *p* = 0.569, *d* = 0.19 (small effect), sex, *χ*^2^(1) = 0.05, *p* = 0.817, φ = −0.04 (small effect), diagnoses, *χ*^2^(5) = 8.62, *p* = 0.125, φc = 0.46 (medium effect), or employment status, *χ*^2^(5) = 1.11, *p* = 0.953, φ = 0.03 (small effect).

### 3.2. Videos

In the ITT analysis all participants together viewed a total amount of *n* = 394 videos and in the PP analysis *n* = 350 videos. The categories with the most views in total were ‘walks’ (ITT: *n* = 128, 32.5%; PP: *n* = 111, 31.7%) and ‘animals’ (ITT: *n* = 126, 32.0%; PP: *n* = 108, 30.9%). The videos with the most views were ‘alpacas’ (ITT: *n* = 35, 8.9%, PP: *n* = 31, 8.9%), ‘rabbits’ (ITT: *n* = 35, 8.9%; *n* = 30, 8.6%), ‘forest walk’ (ITT: *n* = 32, 8.1%; PP: *n* = 27, 7.7%) and ‘beach walk’ (ITT: *n* = 30, 7.6%; PP: *n* = 26, 7.4%). Participants viewed videos with original nature sounds more often (ITT: *n* = 267, 67.8%; PP: n = 240, 68.6%) than videos with relaxation music (ITT: *n* = 127, 32.2%; PP: *n* = 110, 31.4%). After the intervention (T5) participants most often named the videos ‘river walk’ (ITT: *n =* 12, 30.0%, PP: *n =* 12, 41.4%), ‘beach walk’ (ITT: *n* = 12, 30.0%; PP: *n* = 10, 34.5%), and ‘alpacas’ (ITT: *n* = 10, 25.0%; PP: *n* = 9, 31.0%) as one of their best liked videos.

### 3.3. Feasibility

#### 3.3.1. Frequency of use

Most of the *N* = 40 participants in the ITT analysis (*n* = 30, 75.0%) used the VR device in three or 4 weeks of the intervention, *n* = 19 (47.5%) used it in all 4 weeks and *n* = 11 (27.5%) used it in 3 weeks. In the PP analysis a majority of participants (*n* = 18, 62.1%) used the VR device in all 4 weeks and *n* = 11 (37.9%) participants used the VR device three out of 4 weeks. Nearly all participants who finished all questionnaires intended that they used the VR device for the intended two or more times a week (ITT: *n* = 30, 83.3%; PP: *n* = 26, 89.7%).

#### 3.3.2. User-friendliness

Nearly all participants in the ITT and PP analysis described the user-friendliness of the VR device and the application as rather or very easy (ITT: *n* = 33, 91.7%; PP: *n* = 26, 89.7%), while only very few participants described it as rather difficult (ITT: *n* = 3, 8.3%; PP: *n* = 3, 10.3%) and none of them as very difficult. Most participants indicated that they needed less than 3 minutes from turning on the VR device to start watching a video (ITT: *n* = 28, 77.8%; PP: *n* = 24, 82.2%). In the ITT analysis *n* = 7 (19.4%) needed less than 5 min, and only *n* = 1 (2.8%) participant needed more than 10 min. In the PP analysis the remainder of the participants (*n* = 5, 17.2%) indicated that they needed less than 5 minutes.

### 3.4. Satisfaction

In the evaluations at the end of each week (T1–T4), a majority of participants who used the VR device during that week indicated that the videos helped them generally or definitely to relax and/or had a positive benefit on their psychological well-being (ITT: week 1: *n* = 26/36, 72.2%; week 2: *n* = 23/31, 74.2%; week 3: *n* = 28/28, 100.0%, week 4: *n* = 22/27, 81.5%; PP: week 1: *n* = 23/29, 79.3%; week 2: *n* = 21/26, 80.8%; week 3: *n* = 17/25, 68.0%, week 4: *n* = 19/25, 76.0%). Additionally, most participants indicated that they would generally or definitely recommend the intervention with these videos to a friend (ITT: week 1: *n* = 30/36, 83.3%; week 2: *n* = 24/31, 77.4%; week 3: *n* = 20/28, 71.4%, week 4: *n* = 21/27, 76.0%; PP: week 1: *n* = 23/29, 79.3%; week 2: *n* = 20/26, 76.9%; week 3: *n* = 19/25, 76.0%, week 4: *n* = 20/25, 77.8%).

The adapted ZUF-8 had a post-intervention (T5) mean sum score of 19.42 (SD = 4.08) in the ITT analysis and 20.00 (SD = 4.19) in the PP analysis. Most participants in the ITT and in the PP analysis indicated that they would generally or definitely recommend VR to a friend who needed similar help (ITT: *n* = 25, 69.4%; PP: *n* = 24, 82.7%), and that the videos helped somehow or a lot to benefit their psychological wellbeing (ITT: *n* = 28, 77.8%; PP: *n* = 23, 81.3%). In total, a majority of participants indicated that they generally or definitely had a feeling of immersion into the virtual environment (ITT: *n* = 24, 66.7%; PP: *n* = 19; 65.5%). The internal consistency of the ZUF-8 was good, with Cronbach’s alpha = 0.87.

Neither the ITT nor the PP analysis showed a statistically significant difference in levels of satisfaction between women (ITT: 19.73, SD = 4.23; PP: *M* = 20.50, SD = 4.34) and men ITT: *M* = 18.60, SD = 3.75; PP: *M* = 18.89, SD = 3.86; ITT: *t*(34) = 0.74, *p* = 0.464, *d* = 0.28 (small effect); PP: *t*(27) = −0.96, *p* = 0.348, *d* = 0.38 (small effect), and no significant correlation between the participants’ age and satisfaction with the intervention, ITT: *r* = 0.03, *p* = 0.877; PP: *r* = 0.00, *p* = 0.993.

### 3.5. Depressive symptoms and quality of life

The evaluations of the PHQ-9 scores before and after the intervention showed an average level of moderate depressive symptoms in the ITT and PP analysis (see Table 3). The post-intervention mean scores were significantly lower, ITT: *t*(35) = 6.35, *p* < 0.001, *d_z_* = 1.06 (large effect); PP: *t*(28) = 5.99, *p* < 0.001, *d_z_* = 1.11 (large effect), compared to the scores at baseline (T5; see Table 3). At baseline (T0) most participants (ITT: *n* = 29, 72.5%; PP: *n* = 22, 75.9%) reached moderate to severe depressive symptoms, while at post-intervention (T5) most of them showed mild to moderately severe symptoms (ITT: *n* = 30, 75.0%; PP: *n* = 24, 82.8%; see Table 3).

**Table 3:** Results of baseline (T0) and post-intervention (T5) assessments.

Variable	T0 (*n* = 40)	T5 (*n* = 36)	*p*
Intention-to-treat (*N* = 40)			
Depressive symptoms (PHQ-9 scores)			
Minimal: 0–4, *n* (%)	1 (2.5)	3 (8.3)	
Mild: 5–9, *n* (%)	10 (25.0)	13 (36.1)	
Moderate: 10–14, *n* (%)	8 (20.0)	10 (27.8)	
Moderately severe: 15–19, *n* (%)	15 (37.5)	7 (19.4)	
Severe: 20–27, *n* (%)	6 (15.0)	3 (8.3)	
Sum score, *M* (SD)	14.13 (6.18)	10.86 (5.32)	< 0.001
Quality of life (WHOQOL-BREF scores)			
Physical, *M* (SD)	52.58 (19.32)	56.05 (18.05)	0.079
Psychological, *M* (SD)	41.44 (19.47)	47.69 (19.40)	0.005
Social, *M* (SD)	53.70 (17.64)	56.94 (18.31)	0.217
Environmental, *M* (SD)	68.32 (14.35)	69.44 (16.13)	0.432
Credibility and Expectancy (CEQ score)			
Credibility factor, *M* (SD)	19.90 (4.09)	18.48 (5.40)	0.158
Expectancy factor, *M* (SD)	12.93 (3.33)	11.03 (6.10)	0.022
	T0 (*n* = 29)	T5 (*n* = 29)	*p*
Per-protocol (*N* = 29)			
Depressive symptoms (PHQ-9 scores)			
Minimal: 0–4, *n* (%)	1 (3.4)	3 (10.3)	
Mild: 5–9, *n* (%)	6 (20.7)	10 (34.5)	
Moderate: 10–14, *n* (%)	8 (27.6)	9 (31.0)	
Moderately severe: 15–19, *n* (%)	10 (34.5)	5 (17.2)	
Severe: 20–27, *n* (%)	4 (13.8)	2 (69.0)	
Sum score, *M* (SD)	14.03 (6.12)	10.48 (5.12)	< 0.001
Quality of life (WHOQOL-BREF scores)			
Physical, *M* (SD)	52.46 (19.04)	57.27 (17.74)	0.038
Psychological, *M* (SD)	42.67 (18.92)	50.29 (3.45)	0.002
Social, *M* (SD)	55.17 (17.74)	59.47 (18.99)	0.168
Environmental, *M* (SD)	68.43 (2.63)	69.50 (2.95)	0.537
Credibility and Expectancy (CEQ score)			
Credibility factor, *M* (SD)	19.62 (3.96)	19.52 (4.87)	0.898
Expectancy factor, *M* (SD)	13.35 (3.33)	12.35 (6.00)	0.260

Calculation of % from valid cases.

In the ITT analysis and PP analysis the evaluation of the WHOQOL-BREF at baseline (T0) showed on average medium levels of quality of life with highest scores in environmental quality of life and lowest scores in psychological quality of life (see Table 3). At post-intervention (T5), participants in both ITT and PP analyzes, respectively, reached higher scores in all domains of quality of life (see Table 3).

In the ITT analysis psychological quality of life was significantly higher at T5 compared to baseline, *t*(35) = −3.01, *p* = 0.005, *d_z_* = −0.50 (medium effect), while there were no significant differences between T0 and T5 in physical, *t*(35) = −1.81 *p* = 0.079, *d_z_* = −0.30 (small effect), in social, *t*(35) = −1.26, *p* = 0.217, *d_z_* = −0.21 (small effect), or in environmental quality of life, *t*(35) = −0.79, *p* = 0.432, *d_z_* = −0.13 (small effect; see Table 3). In the PP analysis a significant improvement from T0 to T5 was reached in physical quality of life, *t*(28) = −2.18, *p* = 0.038, *d_z_* = −0.41 (small effect), and psychological quality of life, *z* = 3.12, *p* = 0.002, *r* = 0.58 (large effect; see Table 3). However, social quality of life, *z* = 1.38, p = 0.168, *r* = 0.26 (small effect), and environmental quality of life, *t* (28) = −0.63, *p* = 0.537, *d_z_* = 0.12 (small effect), did not differ significantly between T0 and T5 in the PP analysis (see Table 3).

A correlation analysis showed that neither in the ITT nor in the PP analysis there was a significant correlation between depressive symptoms at baseline and satisfaction, ITT: *r* = −0.03, *p* = 0.868; PP: *r* = −0.08, *p* = 0.682. Further there was no significant correlation, between post-intervention (T5) depressive symptoms and satisfaction, ITT: *r* = −0.21, *p* = 0.209; PP: *r* = −0.24, *p* = 0.214.

### 3.6. Credibility and expectancy

At baseline, credibility scores were high in the ITT and the PP analysis, while post-intervention the credibility scores were only high in the PP analysis (see Table 3). In the ITT as well as in the PP analysis mean expectancy scores were moderately low before and after the intervention (see Table 3). The internal consistency of the CEQ at T0 was acceptable, with Cronbach’s alpha = 0.78, and excellent at T5, with Cronbach’s alpha = 0.92.

In the ITT analysis no significant difference between baseline and post-intervention could be found in credibility scores, *t*(35) = −1.44, *p = 0*.158 *d_z_* = 0.24 (small effect), but expectancy scores before the intervention were significantly higher compared to post-intervention, *t*(35) = 2.40 *p* =, *d_z_* = 0.40 (small effect). In the PP analysis there was neither a significant difference in credibility, *t*(28) = 0.13, *p* = 0.898, *d_z_* = 0.02 (small effect), nor in expectancy scores, *t*(28) = 1.15, *p* = 0.260, *d_z_* = 0.21 (small effect) between T0 and T5 (see Table 3).

A correlation analysis showed a statistically significant positive correlation between baseline credibility factor and satisfaction, ITT: *r* = 0.39 (moderate correlation), *p* = 0.019; PP: *r* = 0.48 (moderate correlation), *p* = 0.008, and between baseline expectancy factor and satisfaction, ITT: *r* = 0.53 (strong correlation), *p* < 0.001; PP: *r* = 0.51 (strong correlation), *p* = 0.005.

Additionally, in the PP analysis, a statistically significant negative correlation, could be found between baseline credibility factor and post-intervention outcome of depressive symptoms (PHQ-9), *r* = −0.50 (strong correlation), *p* = 0.006, as well as a significant positive correlation between baseline credibility factor and post-intervention outcome of quality of life (WHOQOL-BREF) regarding physical, *r* = 0.50 (strong correlation), *p* = 0.005, psychological, *ρ* = 0.49 (moderate correlation), *p* = 0.009, and social quality of life, *ρ* = 0.62 (strong correlation), *p* < 0.001. In the ITT analysis a significant positive correlation could only be found between baseline credibility and post-intervention social quality of life, *r* = 0.47 (moderate correlation), *p* = 0.004.

### 3.7. Non-use of the VR intervention

In the evaluation after each week participants who did not use the VR device in at least 1 week (ITT: *n* = 21, 52.5%, PP: *n* = 11, 37.9%) most often chose ‘technical problems like slow internet connection or insufficient video resolution’ (ITT: *n* = 9, 42.9%; PP *n* = 4, 36.4%) and ‘no time’ (ITT: *n* = 5, 23.8%; PP *n* = 4, 36.4%) as their main reasons for non-use of the VR device.

Of the *n* = 10 dropouts, who used the VR device less than 3 weeks, *n* = 4 (40.0%) stopped using the VR device after 1 week, and *n* = 6 (60.0%) after 2 weeks. In the telephone interviews after the intervention, half of the dropouts (*n* = 5, 40.0%) named ‘technical problems like slow internet connection or insufficient video resolution’ as their main reasons for non-use. Only *n* = 2 (30.0%) dropouts stopped using the VR device because they had side effects like dizziness or nausea. The remainder of dropouts indicated that they had either had no time (*n* = 2, 20.0%) or no motivation (*n* = 1, 10.0%) to use the VR device.

## 4. Discussion

### 4.1. Principal findings

In this study we investigated a supportive therapy-accompanying VR relaxation intervention in a psychiatric outpatient setting. All in all, the results of the study indicated that the intervention was successful regarding acceptability and feasibility in this setting, as well as in user-satisfaction.

First, an important finding is that most participants used the VR devices in the recommended way, even if some studies stated that mental disorders could be a prevalent factor for poor compliance (55–57). Despite the participants’ mental instability, a majority of participants in the ITT as well as in the PP analysis used their VR device at least three out of 4 weeks and most participants used the device the recommended two or more times a week. This could be explained by the high user-friendliness, which was rated as rather or very easy by nearly all participants in the ITT and PP analysis. The fact that in the ITT analysis a smaller part of participants used the device three or 4 weeks is caused by the definition of completers and dropouts. It is important to emphasize here that reasons for non-use were not at all a low user-friendliness, but other reasons, that will be described below. After the introduction meeting all participants gained the necessary knowledge to use the VR device independently in their home environment. These findings strongly support our hypothesis that such an intervention is feasible and acceptable in a psychiatric outpatient setting.

It is important to bear in mind that the information about frequency of use, watch duration and video selection was only based on self-report. Since the application was not designed by the researchers, it was not possible to verify this data, which would however be an interesting point for future studies.

Another notable finding is that according to the mean sum score of ZUF-8 the intervention was satisfactory for participants in the ITT and PP analysis. After each week most participants in both analyzes indicated that the VR videos helped them to relax and/or had a positive benefit on their psychological well-being. This is in line with the results of previous research showing that VR can promote relaxation (24, 58, 59) and that relaxation is likely to improve physical and psychological well-being (26, 27, 60–62). In other studies it has been shown before that exposure to real nature outdoors had positive effects on stress and mood levels (63, 64), and that exposure to virtual nature through immersive VR can have similar positive effects on mental health (29, 65, 66). After the intervention approximately 80% of participants in the ITT and PP analysis stated that they would recommend VR to a friend who needed similar help. In both analyzes a majority of participants further indicated that the videos helped somehow or a lot to benefit their psychological wellbeing. According to these results it can be inferred that participants received the support that was intended to be offered, and that in general a VR intervention can be highly satisfactory for patients with severe mental disorders.

Neither in the ITT nor in the PP analysis the participants’ level of satisfaction was related to sex, age, or depressive symptoms at baseline or post-intervention, which shows that VR could be an opportunity for various groups of patients. This is an important finding because patients with mental disorders are mostly presenting a very heterogeneous group. This study also followed a transdiagnostic approach which has been shown to have great potential in psychiatric treatment (67, 68).

The finding that the VR videos helped patients to relax also raises the question which relaxing elements were particularly important. Regarding visual stimuli, results showed that participants particularly enjoyed watching videos in the categories ‘walks’ and ‘animals’, with the three best liked videos being ‘river walk’, ‘beach walk’, and ‘alpacas’. A possible explanation for this preference could be that it might be easier for participants to relax and not get distracted (e.g., by own thoughts) when the environment was changing, as was the case during the walks or when animals were moving around. In future studies it would be interesting to investigate in more detail why certain videos are preferred and during which kinds of videos the highest relaxation is experienced. Further, it would be a really interesting question if specific videos or categories of videos show better improvements in the users’ mental health than others.

Since most of the viewed videos, including the three best liked videos, were accompanied by their original nature sounds, nature sounds could also be considered a particularly relaxing element. It is already known from former studies that exposure to nature sounds can produce positive effects on mood, stress levels and cognition (69, 70).

Another important element benefiting the process of relaxation is the feeling of being present, which is created by the immersive effect of 360-VR (71). In this study a majority of participants in the ITT and PP analysis indicated after the intervention that they generally or definitely had a feeling of immersion into the virtual environment. For many patients with severe mental disorders it is difficult to maintain a certain level of concentration (72) that is required for most other forms of relaxation practices such as meditation or image-guided practices. Therefore, an advantage of VR relaxation interventions could be that due to their immersive nature, these interventions do not place high demands on the ability to concentrate.

In the ITT and PP analysis participants’ moderate level of depressive symptoms at baseline showed a significant decrease after the intervention. Similar outcomes could be observed regarding the post-intervention psychological quality of life in the ITT and PP analyzes, and physical quality of life in the PP analysis, showing significant improvements compared to baseline. In line with this, several studies already showed that the use of VR effectively reduced stress, negative affect, and anxiety (23, 25, 28, 73–75) as well as physical symptoms like pain (34, 76, 77). However, due to the non-controlled and non-randomized study design as well as possible effects of other parallel ongoing treatment of the participants, it cannot be concluded that these improvements of physical and mental health found in this study were solely the effect of the VR intervention.

Improvements in quality of life and depressive symptoms may also have been due to a general lift in governmental restrictions, but it is important to bear in mind that restrictions did not change much during the time of the conduction of the study. Therefore, it is unlikely that a lift in pandemic-related restrictions played a major role in the reported effects. Since in our study we included patients with different mental disorders it would be also interesting in future studies to separately investigate specific effects on psychiatric symptoms in patients with different mental disorders.

On average the credibility of the intervention was high (PP analysis at T0 and T5; ITT analysis at T0) while the pre-and post-intervention expectancy of participants was moderately low. A significant difference between baseline and post-intervention expectancy with a small effect size could only be found in the ITT analysis but not in the PP analysis. One explanation for this could be that participants who dropped out of the intervention due to slow internet connection or other external reasons, and whose expectations may have decreased somehow as a result, were included in the ITT analysis. The results of our study indicated that in the ITT and PP analysis higher baseline credibility and expectancy both correlated with higher outcomes of satisfaction with the intervention. In the PP analysis higher levels of baseline credibility significantly correlated with lower outcomes of depressive symptoms and higher psychological quality of life post-intervention. These results are comparable to those of previous studies, which indicated a strong association between credibility and expectancy of an intervention and its outcomes in various field of physical and mental health (78–82).

Significant differences appeared between the group of completers and dropouts which shows that patients with certain sociodemographic backgrounds might need more support in future study planning. The main reasons for non-use of the VR intervention in the groups of completers and dropouts were technical problems like ‘slow internet connection’ or ‘insufficient video resolution’. It is important to highlight that firstly this an external problem that does not depend on the condition of participant or the content of the intervention and secondly this problem could be solved in future studies and implementations of VR interventions by developing a download option. A successful outcome of the present study is the fact that none of the participants stopped the intervention because they did not manage to use the VR device themselves, so it can still be assumed that the intervention is feasible.

Finally, it is important to mention that most results of the ITT and PP analysis are comparable to each other. Therefore, we can assume that in general the intervention was feasible, satisfactory and acceptable not only for the group of completers, but also for the participants who had an intention to participate in the intervention but did not complete it for various reasons.

### 4.2. Strengths and limitations

It is important to note that this study was not a randomized-controlled trial. Therefore, the results cannot not be considered as a validation of the effectiveness of VR relaxation interventions. This is one of the first examinations of a homebased VR relaxation intervention in psychiatric outpatients and showed that VR is a feasible and acceptable tool for supporting these severely ill patients. These important results can pave the way for further research, especially randomized-controlled trials, on VR related interventions in psychiatric patients.

The relatively small sample size limits the interpretability and generalizability of the results of this study. However, it is important to bear in mind that this is one of the first naturalistic and transdiagnostic studies investigating the feasibility, acceptability, and user-satisfaction of a home-based relaxing VR intervention in a psychiatric outpatient setting, and that results can serve as a basis for further studies.

In proportion to the sample size the number of dropouts in the study could be considered to be medium to high, but since only one comparable study exists at the moment, it remains finally unclear how many dropouts would be realistic. Important to highlight as a strength of the present study is that dropouts had been (initially planned and scheduled) followed up by a telephone interview. Therefore, this study already provides important insights into aspects which might be important for planning future studies in this area. Furthermore, it can be mentioned as a strength that both an ITT and a PP analysis were performed. Thus, we were able to interpret all present results and keep bias in the results as small as possible.

Since severe motion sickness was an exclusion criterion in this study, it could be considered a limitation that there are thus no data on the prevalence of motion sickness in this study’s sample. We decided to exclude those patients because due to the pandemic situation the intervention was conducted in the patients’ home environment, and it was considered too risky that no direct support would have been possible in the case of patients experiencing side effects in the sense of motion sickness. In further feasibility studies it would certainly be interesting to include patients with motion sickness to figure out how VR could be feasible for as many patients as possible.

## 5. Conclusion

The implementation of a supportive therapy-accompanying VR intervention is feasible in a psychiatric outpatient setting. The VR intervention was well accepted and used independently and without relevant difficulties by patients with severe mental disorders. The intervention was highly satisfactory for most participants and may have had a positive influence on their physical and psychological well-being. According to our results, VR as a home-based relaxation tool can be considered as a promising opportunity to support psychiatric outpatients during future lockdown phases as well as an additional component in routine clinical care.

## Data availability statement

The raw data supporting the conclusions of this article will be made available by the authors, without undue reservation.

## Ethics statement

The studies involving humans were approved by Ethics committee of the Medical Faculty of the University of Leipzig. The studies were conducted in accordance with the local legislation and institutional requirements. The participants provided their written informed consent to participate in this study.

## Author contributions

AH: Conceptualization, Data curation, Formal analysis, Writing – original draft. EK: Conceptualization, Data curation, Writing – original draft. SB: Conceptualization, Data curation, Formal analysis, Writing – original draft. CE: Writing – original draft. CR-K: Conceptualization, Data curation, Supervision, Writing – original draft.
