# Corrigendum: Acceptability, feasibility, and user satisfaction of a virtual reality relaxation intervention in a psychiatric outpatient setting during the COVID-19 pandemic

**DOI:** 10.3389/fpsyt.2023.1358379

**Published:** 2024-01-04

**Authors:** Annika Humbert, Elisabeth Kohls, Sabrina Baldofski, Carola Epple, Christine Rummel-Kluge

**Affiliations:** ^1^Department of Psychiatry and Psychotherapy, Medical Faculty, Leipzig University, Leipzig, Germany; ^2^Department of Psychiatry and Psychotherapy, Leipzig University Medical Center, Leipzig, Germany; ^3^Lab E GmbH, Esslingen, Germany

**Keywords:** virtual reality, relaxation, feasibility, psychiatric outpatients, mental

In the published article, there was an error in Table 3 as published. Under the Intention-to-treat section there are missing the four scores of quality of life (WHOQOL-BREF scores) at T0. The corrected Table 3 and its caption appear below.

**Table 3:** Results of baseline (T0) and post-intervention (T5) assessments.

**Variable**	**T0 (*n* = 40)**	**T5 (*n* = 36)**	***p***
**Intention-to-treat (*****N*** = **40)**			
Depressive symptoms (PHQ-9 scores)			
Minimal: 0–4, *n* (%)	1 (2.5)	3 (8.3)	
Mild: 5–9, *n* (%)	10 (25.0)	13 (36.1)	
Moderate: 10–14, *n* (%)	8 (20.0)	10 (27.8)	
Moderately severe: 15–19, *n* (%)	15 (37.5)	7 (19.4)	
Severe: 20–27, *n* (%)	6 (15.0)	3 (8.3)	
Sum score, *M* (*SD*)	14.13 (6.18)	10.86 (5.32)	< 0.001
Quality of life (WHOQOL-BREF scores)			
Physical, *M* (*SD*)	52.58 (19.32)	56.05 (18.05)	0.079
Psychological, *M* (*SD*)	41.44 (19.47)	47.69 (19.40)	0.005
Social, *M* (SD)	53.70 (17.64)	56.94 (18.31)	0.217
Environmental, *M* (SD)	68.32 (14.35)	69.44 (16.13)	0.432
Credibility and expectancy (CEQ score)			
Credibility factor, *M* (SD)	19.90 (4.09)	18.48 (5.40)	.158
Expectancy factor, *M* (SD)	12.93 (3.33)	11.03 (6.10)	.022
	**T0 (*****n*** = **29)**	**T5 (*****n*** = **29)**	***p***
**Per-protocol (*****N*** = **29)**			
Depressive symptoms (PHQ-9 scores)			
Minimal: 0–4, *n* (%)	1 (3.4)	3 (10.3)	
Mild: 5–9, *n* (%)	6 (20.7)	10 (34.5)	
Moderate: 10–14, *n* (%)	8 (27.6)	9 (31.0)	
Moderately severe: 15–19, *n* (%)	10 (34.5)	5 (17.2)	
Severe: 20–27, *n* (%)	4 (13.8)	2 (69.0)	
Sum score, *M* (SD)	14.03 (6.12)	10.48 (5.12)	< 0.001
Quality of life (WHOQOL-BREF scores)			
Physical, *M* (SD)	52.46 (19.04)	57.27 (17.74)	0.038
Psychological, *M* (SD)	42.67 (18.92)	50.29 (3.45)	0.002
Social, *M* (SD)	55.17 (17.74)	59.47 (18.99)	0.168
Environmental, *M* (SD*)*	68.43 (2.63)	69.50 (2.95)	0.537
Credibility and expectancy (CEQ score)			
Credibility factor, *M* (SD)	19.62 (3.96)	19.52 (4.87)	0.898
Expectancy factor, *M* (SD)	13.35 (3.33)	12.35 (6.00)	0.260

Calculation of % from valid cases.

In the published article, there was an error in the text. There were two sentences in the article in which numbers were falsely formatted as references.

First, a correction has been made to the section **2. Materials and methods**, *2.5. Measures*, 2.5.5. Depressive symptoms. This sentence previously stated:

“In addition, sum scores were classified to represent different levels of severity of depressive symptoms from minimal (0–4), mild (5–9), moderate (10–14), moderately severe (15–19) to severe (20–27, 47).”

The corrected sentence appears below:

“In addition, sum scores were classified to represent different levels of severity of depressive symptoms from minimal, 0 to 4, mild, 5 to 9, moderate, 10 to 14, moderately severe, 15 to 19, to severe, 20 to 27 (47).”

Second, a correction has been made to the section **3. Results***, 3.5. Depressive symptoms and quality of life*, paragraph 3. This sentence previously stated:

“However, social quality of life, *z* = 1.38, *p* = 0.168, *r* = 0.26 (small effect), and environmental quality of life, *t* (28) = −0.63, *p* = 0.537, *d*_*z*_ = 0.12 (small effect), did not differ significantly between T0 and T5 in the PP analysis (see Table 3).”

The corrected sentence appears below:

“However, social quality of life, *z* = 1.38, *p* = 0.168, *r* = 0.26 (small effect), and environmental quality of life, *t*(28) = −0.63, *p* = 0.537, *d*_*z*_ = 0.12 (small effect), did not differ significantly between T0 and T5 in the PP analysis (see Table 3).”

In the published article, there was an error in Figure 1 as published. The word “Recriutment” was corrected to “Recruitment”. The corrected Figure 1 and its caption appear below.

**Figure 1:**
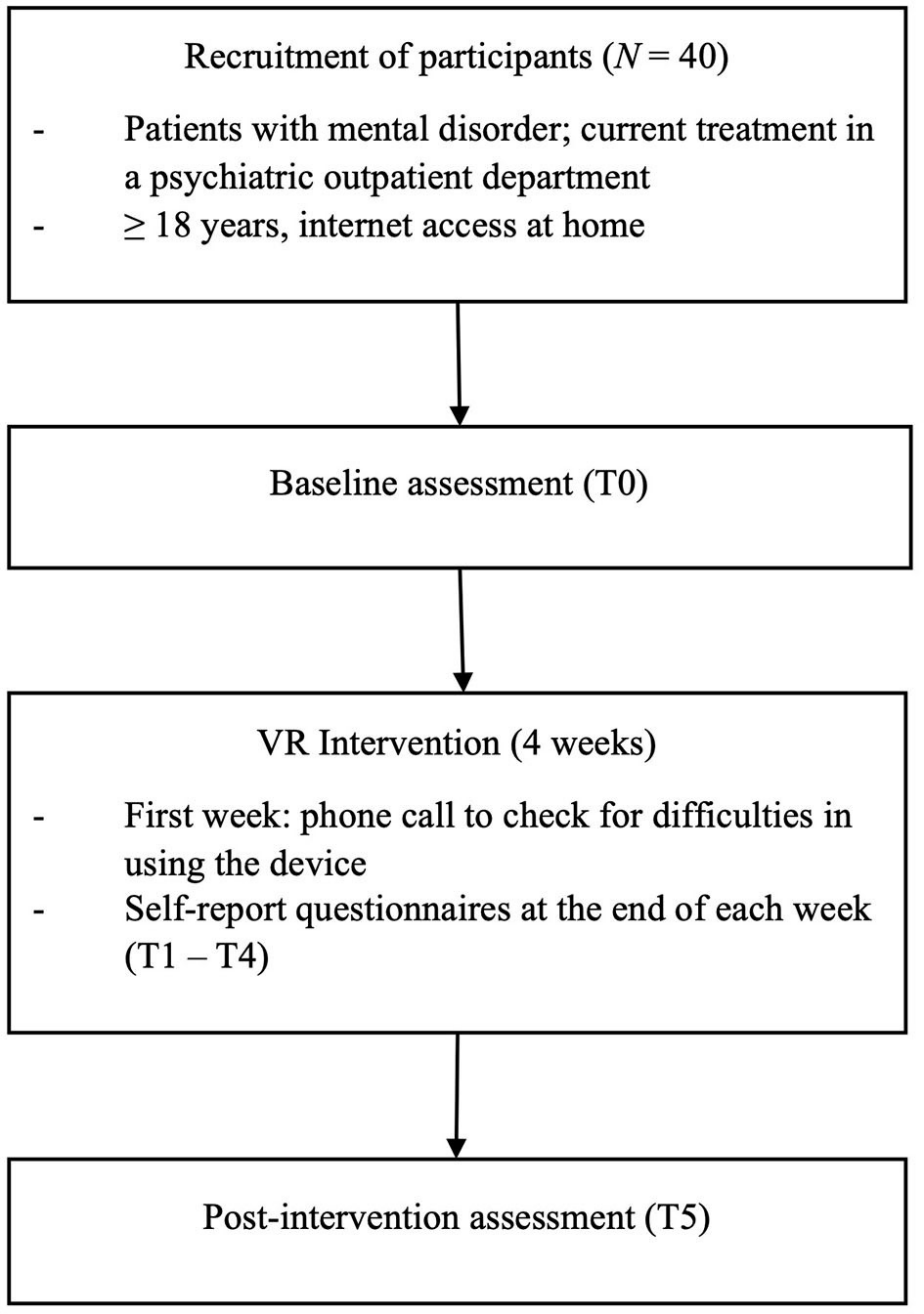
Flowchart of trial design.

The authors apologize for these errors and state that this does not change the scientific conclusions of the article in any way. The original article has been updated.

